# Structural Basis for the Differential Regulatory Roles of the PDZ Domain in C-Terminal Processing Proteases

**DOI:** 10.1128/mBio.01129-19

**Published:** 2019-08-06

**Authors:** Chuang-Kai Chueh, Nilanjan Som, Lu-Chu Ke, Meng-Ru Ho, Manjula Reddy, Chung-I Chang

**Affiliations:** a Institute of Biochemical Sciences, College of Life Science, National Taiwan University, Taipei, Taiwan; b CSIR-Centre for Cellular and Molecular Biology, Hyderabad, India; c Institute of Biological Chemistry, Academia Sinica, Taipei, Taiwan; National Cancer Institute

**Keywords:** PDZ domain, enzyme activation, mechanisms of action, proteases, CTP, Prc

## Abstract

Carboxyl (C)-terminal processing proteases (CTPs) participate in protective and regulatory proteolysis in bacteria. The PDZ domain is central to the activity of CTPs but plays inherently different regulatory roles. For example, the PDZ domain inhibits the activity of the signaling protease CtpB by blocking the active site but is required for the activation of Prc (or Tsp), a tail-specific protease that degrades SsrA-tagged proteins. Here, by structural and functional analyses, we show that in the unliganded resting state of Prc, the PDZ domain is docked inside the bowl-shaped scaffold without contacting the active site, which is kept in a default misaligned conformation. In Prc, a hydrophobic substrate sensor distinct from CtpB engages substrate binding to the PDZ domain and triggers a structural remodeling to align the active-site residues. Therefore, this work reveals the structural basis for understanding the contrasting roles of the PDZ domain in the regulation of CTPs.

## Abstract (precis)

Prc, also known previously as Tsp, is the founding member of the carboxyl-terminal processing protease (CTP) family of PDZ domain-containing proteases that include CtpA and CtpB. The substrate-binding PDZ domain is responsible for regulating the protease activity of CTP proteases; however, the regulatory role of PDZ domain is stimulatory in Prc but inhibitory in CtpA/B. By determining a series of crystal structures of Prc in the unliganded resting state, this study presents the structural basis for PDZ-dependent activation of Prc, the results of which explain the contrasting roles of the PDZ domain in the regulation of the protease activity of CTPs.

## INTRODUCTION

In Gram-negative bacteria, the periplasm is a multifunctional compartment between the porous outer membrane and the inner (or cytoplasmic) membrane. Similar to the eukaryotic endoplasmic reticulum, the bacterial periplasm provides essential functions, including transport, folding, oxidation, and quality control of proteins and lipoproteins; it also confers mechanical strength to the cell by synthesizing the polymeric peptidoglycan (PG), an important structural element of the cell wall (1).

Prc (also named Tsp) is a member of the family of C-terminal processing proteases (CTPs) (2) which features an embedded regulatory PDZ domain inserted into a serine protease domain (3, 4). CTPs are located in the periplasm of Gram-negative bacteria. In Escherichia coli, Prc is involved in degrading abnormal proteins that are unfolded or partially folded and marked cotranslationally at the C terminus by an SsrA tag (5) and that are exported to the periplasm through the Sec translocon (6). Prc is also responsible for C-terminal processing of the lipoprotein penicillin-binding protein 3 (FtsI) (3), a key component of the divisome that catalyzes the cross-linking of PG during cell division (7, 8). By associating with its adaptor protein, NlpI, Prc is also involved in the regulated proteolysis of MepS, a lipid-anchored hydrolase specific for PG cross-links (9). The protease activity of Prc in the periplasm contributes to bacterial evasion of killing by the host complement system (10). Deletion of *prc* results in an altered cell morphology, temperature-sensitive growth under osmotic stress, a reduced heat shock response, the leakage of periplasmic proteins, increased antibiotic sensitivity, and reduced virulence (3, 10–13), which are in accordance with the role of Prc in processing the periplasmic lipoproteins involved in PG synthesis.

The PDZ domain of CTPs is responsible for substrate binding and involved in regulating the activity of CTPs. In the signaling protease CtpB, which forms an intertwined dimeric ring, the PDZ domain plays an inhibitory role by physically blocking the proteolytic active site, thereby disrupting the catalytic triad; deletion of the PDZ domain yields a constitutively active protease (14). Substrate binding activates CtpB by inducing the repositioning of the PDZ domain away from the proteolytic site, which is mediated by a polar amino acid from the PDZ domain as the substrate sensor (14).

In contrast, Prc forms a monomeric bowl-like structure with an attached PDZ domain (4). The activity of Prc strictly requires the PDZ domain, and the PDZ deletion completely abolishes the protease activity (4). Moreover, a pair of hydrophobic residues located in the hinged region connecting to the PDZ domain is proposed to be the substrate sensor mediating the activation of Prc by substrate binding (4). Therefore, the substrate-triggered activation of Prc is likely mediated by the PDZ domain through a structural mechanism distinct from that of CtpB.

To understand the structural basis for the activating role of the PDZ domain in regulating the activity of Prc, we have determined a set of crystal structures in the unliganded resting state of Prc, alone or in complex with NlpI, with a deleted PDZ domain or site-directed mutations in either the PDZ ligand-binding site or the substrate-sensing hinge. In the unliganded resting state, the lid-like PDZ domain is positioned inside the bowl-like body but does not make contact with the proteolytic site. In the structure, the hinge region is deformed into coils and the proteolytic active-site residues are misaligned. A similar inactive conformation of the proteolytic site is also seen in the structures of Prc with the PDZ deletion or mutated hinge residues. Comparison of these structures to the substrate-bound activated structure of Prc reveals how substrate binding to the PDZ domain induces extensive alignment of the proteolytic active-site residues, mediated by structural rearrangement of the substrate-sensing hinge. Overall, these results provide the structural basis for understanding the contrasting regulatory roles of the PDZ domain in the activation of different CTPs.

## RESULTS

### Characterizing Prc with mutations in the substrate-binding sites.

**FIG 1:**
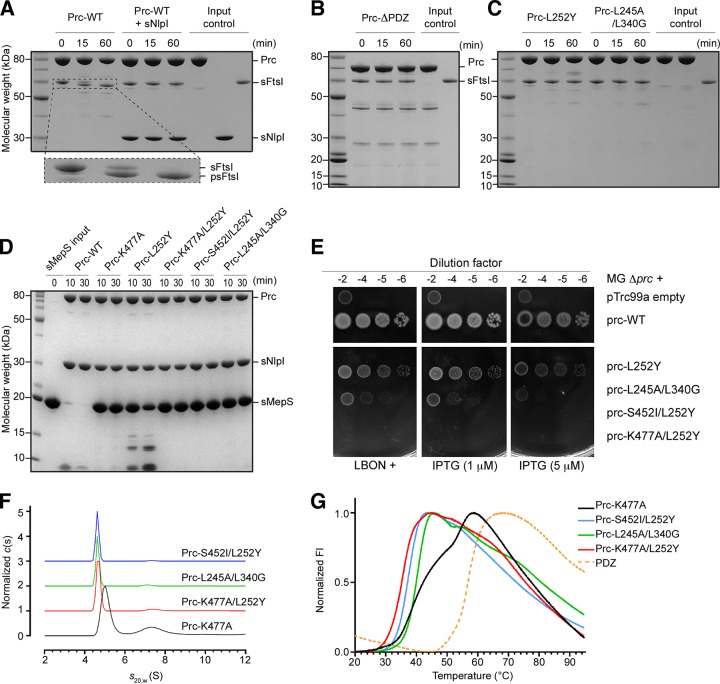
Biochemical and biophysical properties of Prc and various mutants. (A) SDS-PAGE analysis monitoring the cleavage of FtsI missing the N-terminal transmembrane segment (residues 57 to 588; sFtsI) into the processed form (residues 57 to 577; psFtsI) by wild-type Prc with and without NlpI (expressed without the lipid anchor; sNlpI). The dashed area of the gel is enlarged and shown below to highlight the cleavage reaction. (B) Assays of sFtsI cleavage by Prc with the PDZ domain deleted. (C) Assays of sFtsI cleavage by Prc with mutations in the PDZ ligand-binding pocket (L252Y) and the substrate-sensing residues (L245A and L340G). (D) Assays of degradation of MepS, expressed without the lipid anchor (sMepS), by wild-type Prc and various Prc mutants. (E) Viability assays examining the effects of various Prc mutations on the growth of the Δ*prc* mutant (MG Δ*prc*) on medium of low osmotic strength (LBON). Wild-type *prc* in the pTRC99a plasmid has a leaky expression sufficient to complement the Δ*prc* mutant even without the inducer IPTG. (F) Sedimentation velocity profiles comparing the molecular sizes of various Prc double mutants and Prc-K477A, which was in the liganded activated state (4). (G) DSF melting curves comparing the melting transitions of the Prc double mutants with liganded Prc-K477A and the isolated PDZ domain. FI, fluorescence intensity.

In order to capture Prc in the unliganded resting state by preventing substrate binding to the PDZ domain and the proteolytic site, we engineered five Prc mutants, as follows: (i) Prc-ΔPDZ, in which residues 247 to 339 are deleted (4); (ii) Prc-L252Y, which has a mutation designed to block the PDZ ligand-binding pocket; (iii) Prc-K477A/L252Y, which has an additional mutation on the catalytic residue Lys477; (iv) Prc-S452I/L252Y, in which the catalytic residue Ser452 is mutated to isoleucine with a bulky hydrophobic side chain; and (v) Prc-L245A/L340G, which has double mutations on the critical substrate-sensing hinge (4). We next compared the proteolytic activity of these Prc mutants with that of wild-type (WT) Prc against two *in vivo* substrates, FtsI and MepS (3, 9). Using purified recombinant soluble form of FtsI (sFtsI), we showed that it is cleaved by WT Prc into a shorter processed form, supporting previous findings (3) (Fig. 1A). Interestingly, Prc could not cleave sFtsI in the presence of NlpI, which has been shown to be required for the efficient degradation of MepS (Fig. 1A) (4, 9); presumably, the three-sided MepS-docking cradle formed by bound NlpI excludes the access of the larger FtsI to Prc. In contrast, Prc-ΔPDZ, Prc-L252Y, and Prc-L245A/L340G all failed to process sFtsI (Fig. 1B and C). All double Prc mutants also lost MepS-degrading activity (Fig. 1D). Viability assays also showed that none of the double-mutation alleles complemented a *prc* deletion mutant (Fig. 1E). Finally, analytical ultracentrifugation (AUC) analysis indicated that the double mutants had a smaller particle size than Prc-K477A, which is locked in the substrate-bound activated open conformation (4) (Fig. 1F), suggesting that these double mutants may adopt a more compact structure than Prc-K477A. Indeed, thermal shift assays showed single melting transitions for these double mutants distinct from the melting transition for the two-phase Prc-K477A (Fig. 1G).

### Structure of Prc-S452I/L252Y in the unliganded resting state.

**FIG 2:**
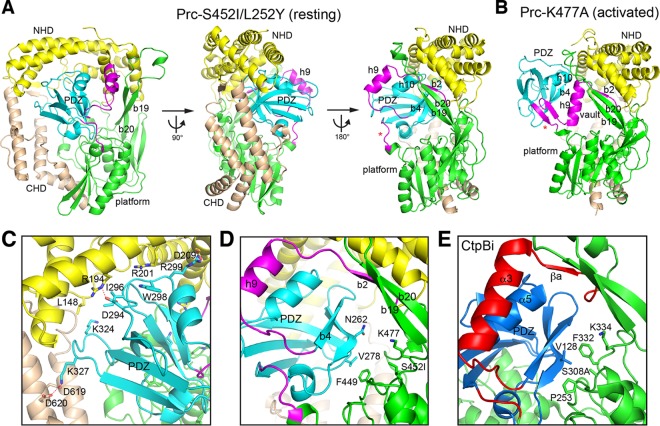
Overall structure of Prc in the resting state. (A) Three orthogonal views of Prc-S452I/L252Y in ribbon representations. (B) For comparison, the structure of Prc-K477A (bound substrates were omitted) determined in the activated state (4) was shown in the same orientation as the right view in panel A. (C and D) Zoom-in views showing the interaction of the PDZ domain with NHD and CHD (C) and with the protease platform (D). (E) Zoom-in view showing the PDZ domain interaction in the inhibited resting CtpB (CtpBi). The associated N- and C-terminal helical domains (NHD and CHD, respectively) are shown in yellow and wheat, respectively. The PDZ domain is in cyan (Prc) or deep blue (CtpB). The protease domain (platform) is indicated and shown in green. The vault element, consisting of helix h9 and strand b2, and the hinge coil (indicated by an asterisk in Fig. 2A and B) undergoing remodeling during ligand-dependent activation are highlighted in magenta (Prc) or red (CtpB).

According to the results presented above, we performed crystallization screening experiments for each of the Prc double mutants, which likely adopt an unliganded resting-state conformation when they are alone or in complex with NlpI. We determined the structure of Prc-S452I/L252Y (see Table S1 in the supplemental material), which is indeed trapped in the unliganded resting state. Prc has a platform-like protease domain harboring the proteolytic groove, which, in the activated state, is enclosed by a vault-like structural element comprising helix h9 and a three-stranded b2-b19-b20 antiparallel β-sheet (4). In addition to the conserved protease domain, Prc contains extended N-terminal and C-terminal helical domains (named NHD and CHD, respectively), which are joined together via two β-strands (Fig. 2A). In Prc, the vaulted protease domain, NHD, and CHD form a round bowl-like structure. The PDZ domain is inserted into the protease domain between helix h9 and the platform (Fig. 2A and B). In the structure of Prc-S452I/L252Y, the PDZ domain is docked inside the bowl (Fig. 2A), interacting with residues, mainly from NHD and CHD, dispersed across a wide region (Fig. S1A); these residues constitute a total interface area of 1,595 Å^2^. Almost half (49.4%) of the surface residues of the PDZ domain interacting with the bowl are distributed evenly across the surface (Fig. S1B).

10.1128/mBio.01129-19.1 FIG S1 Distribution of the interacting residues of the PDZ domain and the bowl-like scaffold of Prc in the resting state. (A) A view of the bowl-like structure of Prc-S452I/L252Y, highlighting the residues (in blue) interacting with the PDZ domain (omitted for clarity). The coloring scheme is the same as that described in the legend to Fig. 2. (B) The residues of the PDZ domain that interact with the bowl-like body of Prc in the resting state are colored in red in a ribbon model. Download FIG S1, TIF file, 1.1 MB. Copyright © 2019 Chueh et al. 2019 Chueh et al. This content is distributed under the terms of the Creative Commons Attribution 4.0 International license.

10.1128/mBio.01129-19.6 TABLE S1 Data collection and refinement statistics. ^a^, the values for the highest-resolution shell are shown in parentheses; ^b^, *R*_pim_ is the precision-indicating merging *R*, which describes the accuracy of the averaged measurement (37). Download Table S1, DOCX file, 0.03 MB. Copyright © 2019 Chueh et al. 2019 Chueh et al. This content is distributed under the terms of the Creative Commons Attribution 4.0 International license.

### Intramolecular interaction of the docked PDZ domain in Prc.

In the resting structure, the PDZ domain is surrounded by NHD, CHD, and the protease domain. Most of the contacting residues of the PDZ domain are from the loops and are polar amino acids. The interacting residues of NHD and CHD are mostly polar amino acids from the helices and several hydrophobic residues from the loops. However, few are involved in specific side chain interactions, except for the polar pairs Lys327-Asp619, Arg299-Asp209, and Arg194-Asp294 (Fig. 2C). Instead, many residues engage a side chain stacking interaction to form Van der Waals contacts. Perhaps owing to the nonspecific nature of the docking interaction, the PDZ domain in the resting structure shows higher temperature factor (B-factor) values than the bowl-shape scaffold of Prc.

Interestingly, the docked PDZ domain makes little contact with the protease domain; the only contact is made between the stacking side chains of PDZ Val278 and Phe449 at the back, and this contact anchors the PDZ domain in a specific position to expose the ligand-binding strand b4 (Fig. 2D). However, in CtpB, helix α5 of the inhibitory PDZ domain packs against helix α3 and strand βa. Importantly, the PDZ residue Val128 blocks the catalytic Ser308 by binding to a hydrophobic pocket walled by Pro253 and Phe332 (Fig. 2E), but the corresponding PDZ residue in Prc, Asn262, makes no contact whatsoever (Fig. 2D). Therefore, the lack of an extensive interaction of the PDZ domain with the protease domain and of any direct contact of the PDZ domain with the proteolytic active site in the resting structure of Prc is significantly different from the findings for CtpB, which further supports the suggestion that the PDZ domain of Prc does not assume an inhibitory function (4).

### Structural difference between liganded activated Prc and unliganded resting Prc.

**FIG 3:**
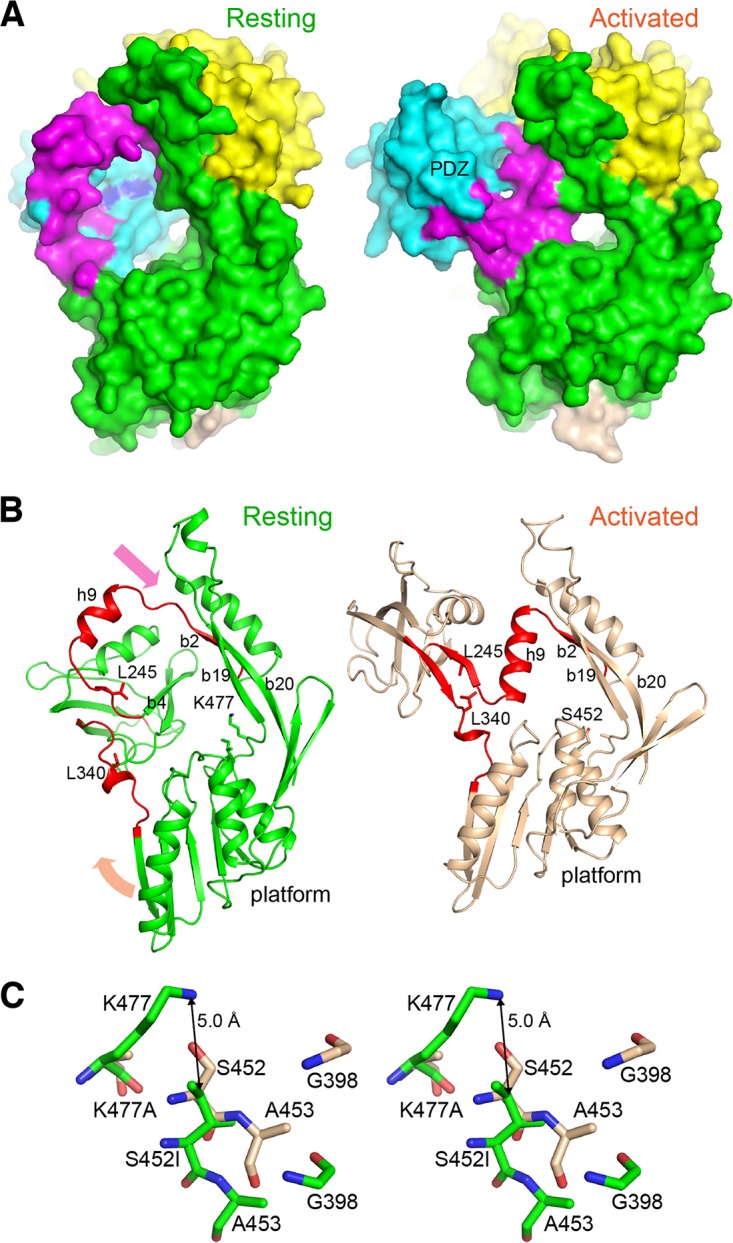
Structural difference between the resting and activated states of Prc. (A) The structures of Prc-S452I/L252Y (left) and Prc-K477A (right; the bound substrate is omitted) are shown in surface representations in a similar orientation. The coloring scheme is the same as that described in the legend to Fig. 2. The PDZ ligand-binding site is highlighted by coloring L252Y in blue. (B) Structural comparison of the resting and activated Prc, highlighting the remodeling of the two hinge coils (red) into a pair of β-strands during activation; the arrows indicate the directions of movement of helix h9 and the protease platform. NHD and CHD were removed for clarity. (C) Stereo view of the catalytic residues Lys477 and Ser452 and the oxyanion hole residues Gly398 and Ala453 in the resting and activated states, shown in sticks in green and wheat, respectively. The superposition was obtained by structural alignment of Prc-S452I/L252Y (chain A; resting form) and Prc-K477A (chain C of PDB accession number 5WQL; activated form) (RMSD, 2.14 Å; 503 residues aligned).

Compared to the structure of the liganded activated state, in the structure of the unliganded resting state, the docked PDZ domain is repositioned to expose its ligand-binding site for the substrate C terminus to an open space above the platform (Fig. 3A). The two-β-stranded substrate-binding hinge, which is formed in the activated state and which connects the PDZ domain to helix h9 and the platform, is unfolded into two coils; the critical substrate-sensing residues Leu245 and Leu340 are separated and become solvent exposed (Fig. 3B). Helix h9 is drifted away and partially unfolded to make up the large vaulted space above the platform (Fig. 3B). Lastly, without a folded connecting hinge, the proteolytic platform is angled down with misaligned active-site residues: the catalytic Lys477 and Ser452 are apart from each other, and the amide groups of Ala453 and Gly398, forming the oxyanion pocket, are out of place (Fig. 3B and C).

### Structures of Prc without the PDZ domain or the hydrophobic sensor.

To assess the structural role of the PDZ domain and the hydrophobic sensor residues, which have been shown to be required for Prc activity, we also determined the crystal structures of Prc-ΔPDZ and Prc-L245A/L340G when both are in complex with NlpI. In both structures, helix h9 and the hinge region are disordered and invisible from the electron density map. Additionally, the entire PDZ domain in the structure of Prc-L245A/L340G is also missing in the electron density map. Nevertheless, the structures of the bowl-like body of Prc-ΔPDZ and Prc-L245A/L340G, including the protease platform, are superimposable with the structure of the resting Prc-S452I/L252Y (Fig. S2A and Table S2). The proteolytic active-site residues in Prc-ΔPDZ and Prc-L245A/L340G are also in the misaligned resting-state conformation (Fig. S2B). These structures confirm that, without the PDZ domain or the hydrophobic substrate sensor, Prc is maintained in the inactive resting state.

10.1128/mBio.01129-19.2 FIG S2 Structures of Prc without the PDZ domain or the hydrophobic sensor. (A) The structures of NlpI–Prc-ΔPDZ and NlpI–Prc-L245A/L340G (NlpI is omitted for clarity) are superimposed on the structure of Prc-S452I/L252Y. The chain breaks owing to the disordered helix h9 and the PDZ domain, which are missing in the electron density map, are marked by red asterisks. (B) Zoom-in window showing the separated catalytic Lys477 and Ser452 residues. Download FIG S2, TIF file, 1.7 MB. Copyright © 2019 Chueh et al. 2019 Chueh et al. This content is distributed under the terms of the Creative Commons Attribution 4.0 International license.

10.1128/mBio.01129-19.7 TABLE S2 Alignment of the resting state structures of Prc. *, the backbone RMSD (in angstroms) was calculated over the indicated chain in each crystal structure and chain A in the structure of Prc-S452I/L252Y with and without outlier rejection (5 cycles; cutoff, 2.0 Å). Download Table S2, DOCX file, 0.02 MB. Copyright © 2019 Chueh et al. 2019 Chueh et al. This content is distributed under the terms of the Creative Commons Attribution 4.0 International license.

### Flexibility of helix h9 in the resting state.

**FIG 4:**
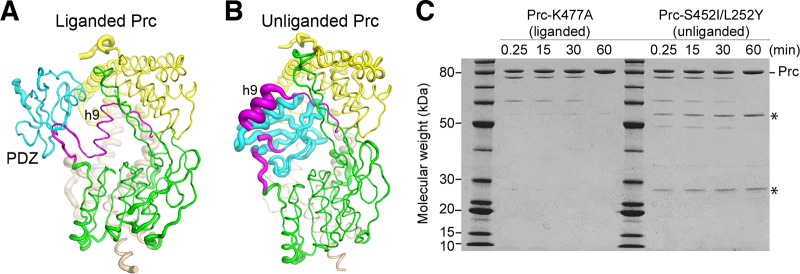
Difference in the structural flexibility of helix h9 in the liganded and unliganded forms. (A and B) Liganded NlpI–Prc-K477A (A) and unliganded NlpI–Prc-S452I/L252Y (B) cocrystallized with NlpI in the same space group in tube representations, with the tube diameter being scaled according to the B factor of the structure. The bound NlpI dimer was omitted for clarity. The coloring scheme is the same as that described in the legend to Fig. 2. (C) SDS-PAGE analysis monitoring the cleavage of Prc by V8 protease at the indicated time points. The two fragments generated by cleavage at residue Asn211 of helix h9 are indicated by asterisks.

We also crystallized Prc-S452I/L252Y bound to NlpI in the space group of P2_1_2_1_2_1_, which differs from that of Prc-S452I/L252Y alone (P3_2_21) but which is the same as that of the substrate-bound NlpI–Prc-K477A crystals trapped in the activated state (4). The crystal contacts of these P2_1_2_1_2_1_ forms involve the NlpI dimer and the NHD and CHD of Prc only; hence, they provide further information about the structural difference between the two conformational states. The structure of Prc-S452I/L252Y in the NlpI-bound complex shows a similar resting-state conformation, with the root mean square deviation (RMSD) being 0.62 by comparison to the structure of Prc-S452I/L252Y alone (Table S2). Interestingly, unlike the liganded Prc (Fig. 4A), the NlpI-complexed Prc-S452I/L252Y shows a flexible helix h9 with a poor electron density map, and the coiled region connecting to the PDZ domain is partially disordered (Fig. 4A). Moreover, the docked PDZ domain in these resting structures has higher B-factor values than the protease domain, which is in sharp contrast to the findings for the liganded activated structure showing a relatively rigid PDZ domain, helix h9, and the substrate-sensing hinge (Fig. 4A and B). To probe the flexibility of h9 in these resting-state mutants in solution, we performed limited proteolysis using V8 protease, which has been shown to cleave Prc at the peptide bond between Asn211 and Thr212 of helix h9, yielding two fragments with molecular masses of 49.5 and 25.7 kDa (15). We found that limited V8 proteolysis indeed resulted in the two fragments from Prc-S452I/L252Y but not from Prc-K477A at various incubation times (Fig. 4C), supporting the flexibility of helix h9 in the resting state.

## DISCUSSION

**FIG 5:**
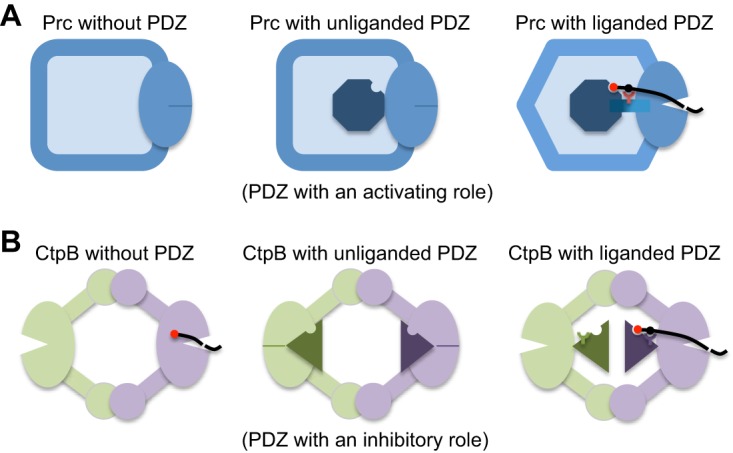
Comparison of the structural mechanisms for the regulation of the protease activity of Prc and CtpB by the PDZ domain. (A) In Prc, the PDZ deletion results in an inactive protease (left), as evidenced by a misaligned protease active site (indicated by a solid dash). In the resting state, the unliganded PDZ domain (the octagon) docks inside the bowl-like scaffold structure of Prc and makes no contact with the protease active site (middle). Upon substrate binding, the hydrophobic sensor (Leu340; indicated by a Y-shaped symbol) engages the bound substrate and triggers structural remodeling to align the protease active site for substrate cleavage (right). (B) In dimeric CtpB, deletion of the PDZ domain yields a constitutively active protease (left). In the inhibited resting state, the protease active site is disrupted by the docked PDZ domain (triangles) (middle). Substrate binding induces repositioning of the PDZ domain, stabilized by the polar sensor (Arg168) from the PDZ domain (right).

The work presented here has revealed the structural basis for the activating role of the PDZ domain in Prc. Our results show that in the unliganded resting state, Prc forms an inactive structure characterized by a deformed proteolytic groove resulting from misalignment of the loops forming the catalytic Lys-Ser dyad and the oxyanion pocket. In the resting state, the PDZ domain of Prc is docked inside a bowl-like scaffold with the ligand-binding site exposed. Notably, the PDZ domain engages in an intramolecular interaction mainly with NHD and CHD rather than with the catalytic active site in the protease domain. In the absence of the PDZ domain, Prc-ΔPDZ also adopted the resting-state structure with a similarly misaligned proteolytic domain. These results support the finding that the PDZ domain regulates the protease activity of Prc by serving as an activator but not an inhibitor. Prc activation is achieved by substrate binding to the PDZ domain, which is sensed by conserved hydrophobic residues Leu340 and Leu254 to stabilize the substrate-bound active conformation. The repositioned PDZ domain induces extensive remodeling of the functional proteolytic platform to enable a substrate cleavage reaction (Fig. 5A).

The mechanism of protease activation by the PDZ domain in Prc shown here is fundamentally different from that in CtpB reported previously (14). In CtpB, the protease is active without the PDZ domain. In the resting state, the PDZ domain of CtpB binds to the protease domain and physically disrupts the catalytic active site. Binding of the substrate to the PDZ domain is sensed by PDZ residue Arg168, which stabilizes the reposition of the PDZ domain away from the protease domain to relieve the inhibition effect (Fig. 5B).

The different roles of the PDZ domain in the activation of Prc and CtpB may be paralleled by those for the structurally unrelated HtrA-family proteases DegP and DegS, in which oligomerization involving the PDZ domains contributes additional regulatory roles (16). DegP requires the PDZ domain for ligand-dependent activation (17), but the PDZ domain in DegS mainly inhibits the activity (18–21). Therefore, the PDZ domain can inherently bring different effects to regulation of the activity of the PDZ-containing proteases.

Many of the structural features of the unliganded Prc and the conformational changes induced by the C terminus of the substrate are also different from those of CtpB. Helix α3 in CtpB, equivalent to helix h9 in Prc, is structured in both the resting and the activated states. In the two states of CtpB, the PDZ domain maintains critical contact with helix α3 by the sensor Arg168 (14). In contrast, the PDZ domain of Prc does not interact with helix h9 in the resting state. Notably, as helix α3 is not partially unfolded in resting CtpB, the open gate framed by the helix in CtpB is not as large as that in the unliganded Prc; interestingly, a structure of CtpA determined in the inactive state shows an even smaller gate (see Fig. S3 in the supplemental material) (22). It is conceivable that the large open gate in Prc, framed by the disordered helix h9, may allow the access of various folded or unfolded substrates with different unstructured C-terminal tails to the more exposed PDZ ligand-binding site. In contrast, CtpA and CtpB are each known to recognize and process one specific substrate (SpoIVFA and photosystem II D1 protein, respectively), explaining their smaller open gates in the resting state. Lastly, CtpB forms a pseudosymmetric ring-like homodimer locked via the N- and C-terminal dimerization domains, which may permit only a limited conformational change triggered by the liganded PDZ domain if only one of its two interlocked subunits is activated by a substrate. However, given the primary inhibitory role of the PDZ domain in CtpB, a substrate-triggered release of the PDZ domain from one catalytic active site may be sufficient to activate CtpB without the need for a larger conformational change of the PDZ and the protease domains shown in the activation of Prc (4).

10.1128/mBio.01129-19.3 FIG S3 Structural differences of the open gate of Prc and the C-terminal processing proteases CtpA and CtpB in the resting state. Surface representations of Prc, CtpB, and CtpA in a similar orientation are shown. The conserved protease domain and the PDZ domain are colored in green and cyan, respectively. The ligand-binding site in the PDZ domain is highlighted in blue. The gate helix h9 of Prc, the corresponding helix in the other CTPs, and the linkers connecting the PDZ domain are in magenta. The nonconserved region is in gray. Download FIG S3, TIF file, 2.7 MB. Copyright © 2019 Chueh et al. 2019 Chueh et al. This content is distributed under the terms of the Creative Commons Attribution 4.0 International license.

Our crystallographic results also reveal that Prc-L245A/L340G, which does not contain the two ligand-sensing residues, has a flexible PDZ domain missing in the electron density map; importantly, the bowl adopts a resting-state structure showing a dislocalized protease platform and deformed active-site loops which are superimposable on the structure of unliganded Prc-S452I/L252Y (Fig. S2A). Since this mutant contains normal PDZ ligand-binding and catalytic residues but has lost completely the ability to degrade MepS or process FtsI, its structure demonstrates that the two hydrophobic residues are important substrate sensors essential for stabilizing the ligand-bound activated conformation of Prc.

Unexpectedly, our structural and limited proteolysis results showed that helix h9 of Prc is flexible in the resting state but becomes structurally defined to narrowly enclose the bound cleavage-site substrate polypeptide in the activated state. The disorder-to-order transition of helix h9, which completes the active site, regulated allosterically by the liganded PDZ domain, is therefore different from that of helix α3 of CtpB but similar to that of the active-site loops of HtrA proteases, such as DegP and DegS (16). However, the active site of HtrA proteases is exposed and does not enclose the substrate. Moreover, the substrate of DegS does not contain a PDZ-binding degron (20); although the substrate of DegP may contain covalently linked cleavage-site and PDZ-binding degrons, they are likely to bind to the active site and the PDZ1 domain, respectively, belonging to different subunits of the oligomeric DegP (23). In contrast, binding of the C terminus of a substrate to the PDZ domain through the large open gate of Prc must always result in the entrapment of the same substrate polypeptide at the active site enclosed by helix h9. Therefore, the activation mechanism of Prc combines features from the activation mechanisms of the CtpB and HtrA proteases.

Finally, our structural studies have provided mechanistic insight into the operation of Prc by the activating PDZ domain undergoing relocation upon substrate binding (Movie S1). During activation, helix h9 moves in a direction opposite that of the liganded PDZ domain and assumes a center position between the two substrate-binding sites in the PDZ domain and the proteolytic groove (Fig. S4A and B). Concurrently, the flexible helix h9 undergoes its own remodeling and a disorder-to-order transition, which serve to completely enclose the substrate polypeptide bound to the proteolytic groove (Fig. 4C). As such, helix h9 may have a pulley-like function to convert the conformational change of the substrate-bound PDZ domain into driving substrate translocation for Prc to degrade folded or incompletely folded protein substrates in *cis*.

10.1128/mBio.01129-19.4 FIG S4 The pulley-like helix h9 in the unliganded resting and the liganded activated states. (A and B) Top views of the unliganded resting Prc-S452I/L252Y (A) and substrate-bound activated Prc-K477A (B). NHD has been removed for clarity. The coloring scheme is the same as that described in the legend to Fig. 2. The bound polypeptide chains are shown in spheres. The dashed curve depicts a plausible connection between the cleavage-site (left) and C-terminal (right) peptides bound to Prc-K477A. (C) Cartoons illustrating the disorder-to-order transition of helix h9 and its pulley-like role associated with the in *cis* substrate-triggered mechanical operation of Prc. The dashed line on the gray substrate C-terminal tail denotes a potential cleavage site (left), which is translocated to the active site and cleaved (denoted by the solid blue line) by the coordinated movements of helix h9 and the liganded PDZ domain in the activated state (right). Download FIG S4, TIF file, 1.2 MB. Copyright © 2019 Chueh et al. 2019 Chueh et al. This content is distributed under the terms of the Creative Commons Attribution 4.0 International license.

10.1128/mBio.01129-19.5 MOVIE S1 Ligand-triggered motion and activation of Prc. The movie presents a sequence showing the overall structure of unliganded Prc, its association with NlpI, and the conformational change, induced by binding of the substrate C terminus to the PDZ domain, from the unliganded resting state (this work; chain A of PDB accession number 6IQR) to the substrate-bound activated state (chain C of PDB accession number 5WQL). Also shown in the sequence is a modeled substrate C-terminal peptide (LSRS), derived from the substrate MepS (4). Helix h9 is in magenta. The PDZ domain is in yellow. The substrate sensor (Leu340) and the catalytic dyad (Lys477 and Ser452) residues are shown as spheres. The movie and morphing were created with the UCSF Chimera system (36). The morphing speed was arbitrarily chosen for illustrative purposes. Download Movie S1, MOV file, 16.2 MB. Copyright © 2019 Chueh et al. 2019 Chueh et al. This content is distributed under the terms of the Creative Commons Attribution 4.0 International license.

## MATERIALS AND METHODS

### Construction of the E. coli BL21(λDE3) *prc*::Cm mutant.

P1 phage-mediated transduction was performed as described previously (38). P1 phage lysate was generated using the MG1655 *prc*::Tn*10*dCm strain, and the *prc*::Tn*10*dCm mutation was transferred by P1 transduction into E. coli BL21(λDE3) and selected on chloramphenicol-containing LB plates (15 μg/ml). This mutant behaves identically to the *prc* deletion mutant.

### Cloning and mutagenesis.

The Prc mutants (Prc-K477A, Prc-L252Y, Prc-S452I, Prc-K477A/L252Y, Prc-S452I/L252Y, Prc-L245A/L340G) were generated by PCR-based site-directed mutagenesis (the primers used are listed in Table S3 in the supplemental material), using a wild-type Prc plasmid as the template (4). Prc-ΔPDZ (Prc with the deletion of residues 247 to 339) with a C-terminal His tag was also cloned into the pTrc99A vector. The DNA sequences encoding the soluble forms of NlpI (sNlpI) and MepS (sMepS), consisting of NlpI and MepS without lipoprotein signal peptides, were cloned into the pET28a and pET21a vectors, respectively. sMepS was cloned with a C-terminal His tag, while sNlpI was expressed with an N-terminal His tag and a tobacco etch virus (TEV) protease cleavage site. All of the constructs were sequenced before follow-up experiments. MG1655 genomic DNA was used as a template to PCR amplify the *ftsI* gene encoding residues 57 to 588 without the N-terminal transmembrane helix (sFtsI) (Table S3). The amplified product and pET28a vector were digested with the NheI and BamHI restriction enzymes (New England Biolabs, USA), purified, and ligated using T4 DNA ligase (New England Biolabs, USA). The positive clones were identified by colony PCR and confirmed by sequence analysis.

10.1128/mBio.01129-19.8 TABLE S3 Primer sequences used in the study. Download Table S3, DOCX file, 0.02 MB. Copyright © 2019 Chueh et al. 2019 Chueh et al. This content is distributed under the terms of the Creative Commons Attribution 4.0 International license.

### Protein expression and purification.

To prevent contamination or preprocessing by endogenous Prc, Prc mutant proteins and sFtsI were expressed in E. coli ΔPrc cells [strains MR812 and BL21(λDE3) *prc*::Cm, respectively] (4). Full-length Prc, sNlpI, and sMepS were expressed in E. coli BL21(λDE3) cells as described previously (4). Cells were grown in LB medium until the optical density at 600 nm reached 0.6 to 0.8 and were induced with 1 mM isopropyl β-d-thiogalactopyranoside (IPTG) for 4 h at 22°C. The cell pellets were collected after centrifugation and resuspended in lysis buffer containing 50 mM Tris-HCl (pH 8.0) and 500 mM NaCl. After being ruptured with a French press (Avestin) and centrifuged at 35,000 × *g*, the supernatants were collected and incubated with Ni-nitrilotriacetic acid resins (Qiagen) for 2 h at 4°C. Proteins were further washed with a stepwise imidazole gradient and eluted with 250 mM imidazole.

All of the recombinant proteins were dialyzed against the different buffer components to remove the imidazole. For further assays, Prc and sNlpI were dialyzed against buffer containing 20 mM Tris-HCl (pH 8.0) and 150 mM NaCl. sMepS was dialyzed against the same buffer components with the addition of 2 mM dithiothreitol (DTT). sFtsI, on the other hand, was dialyzed against buffer containing 20 mM HEPES (pH 7.0) and 150 mM NaCl. Prc and sNlpI were further purified by Mono Q 5/50 GL column chromatography (GE Healthcare) at pH 8.0. For protein crystallization, most of the Prc mutants (Prc-K477A/L252Y, Prc-S452I/L252Y, Prc-PDZ) and sNlpI were first dialyzed against buffer containing 25 mM Tris-HCl (pH 8.0) and 50 mM NaCl. Prc-L245A/L340G was dialyzed against buffer containing 25 mM Tris-HCl (pH 8.0) and 150 mM NaCl after 250 mM imidazole elution. All of the recombinant proteins were then purified by Mono Q 5/50 GL column chromatography at pH 8.0. After that, Prc and sNlpI were concentrated, mixed in a 1:2 molar ratio, and subjected to chromatography on a Superdex 200 10/300 GL column (GE Healthcare), which was equilibrated with buffer containing 25 mM Tris-HCl (pH 8.0) and 150 mM NaCl, to get Prc-NlpI complexes.

### Crystallization and data collection.

The hanging-drop vapor diffusion method was performed for crystallization. Protein solutions with concentrations ranging from 10 to 20 mg/ml were mixed with equal volumes of reservoir solutions, and for most of the protein samples, crystallization experiments were performed at 22°C; the exception was for the sNlpI–Prc-ΔPDZ complex, which was performed at 16°C. For the sNlpI–Prc-L245A/L340G complex, crystals were grown with solutions containing 0.2 M sodium citrate tribasic dihydrate and 10 to 13% polyethylene glycol (PEG) 3350. For the sNlpI–Prc-S452I/L252Y complex, protein was crystallized with a solution containing 0.1 M imidazole (pH 8.0), 0.2 M calcium acetate, and 11% PEG 8000. For Prc-S452I/L252Y, crystals were obtained in a solution containing 0.2 M sodium thiocyanate (pH 6.4 to 6.8) and 20% PEG 3350. For the sNlpI–Prc-ΔPDZ complex, the crystal was grown in a solution containing 0.2 M ammonium sulfate, 0.1 M Tris-HCl (pH 8.5), and 25% PEG 3350. The crystals were cryoprotected by transferring them to their corresponding reservoir solutions supplemented with 20% glycerol or 20% ethylene glycol before data collection. The data set for sNlpI–Prc-L245A/L340G was collected at BL-1A of the Photon Factory (Japan), and the data sets for the other four protein complexes were collected at NSRRC (Taiwan, Republic of China). The diffraction data for sNlpI–Prc-S452I/L252Y and sNlpI–Prc-ΔPDZ were collected at beamline TPS 05A, whereas the data set for Prc-S452I/L252Y was collected at beamline TLS 15A1. All diffraction data were indexed, integrated, and scaled using HKL2000 (24).

### Structure determination and refinement.

Using the structures of sNlpI and separate domains of Prc-K477A (PDB accession number 5WQL) as search models, the complex structure of sNlpI–Prc-S452I/L252Y was solved by molecular replacement with the program Phaser (25). Partial solutions for these tetramer complexes from which the PDZ domain was missing were then obtained and subjected to rigid-body refinement. The final structures were built with the MOLREP program, using two PDZ domains as search models and the difference Fourier maps as the search space (26). The structures of Prc-S452I/L252Y, sNlpI–Prc-L245A/L340G, and sNlpI–Prc-ΔPDZ were solved by molecular replacement with the Phaser program, using the structure of sNlpI–Prc-S452I/L252Y as the search model. The crystals of Prc-S452I/L252Y were found to be merohedrally twinned with a twinning fraction of 0.42, as determined by the Phenix tool Xtriage and as subsequently checked by the CCP4 program TRUNCATE (27, 28). The CCP4 program DETWIN was then used to detwin the data (29, 30). After automated model building using the Phenix tool AutoBuild and the CCP4 program Buccaneer (27, 31), all models were manually adjusted using the Coot program (32) and then iteratively refined with REFMAC5 in the CCP4 package (33). The final models were validated with the programs MolProbity and PROCHECK (34, 35). Crystallographic and refinement statistics are listed in Table S1.

### Degradation assays.

For sFtsI degradation assays, each reaction mixture (10 μl) contained 2 μg WT Prc or a Prc variant and 0.5 μg of sFtsI in buffer containing 20 mM Tris-HCl (pH 8.0) and 150 mM NaCl. Additional sNlpI was added to the Prc WT-sNlpI group in a molar ratio of 1:1 with Prc. All of the reaction mixtures were incubated at 37°C, and the reactions were stopped at the indicated time points by adding 5× SDS-PAGE loading dye. The samples were heated at 98°C and loaded onto 4 to 12% bis-Tris gels (Invitrogen). Protein bands were then detected by Coomassie blue staining. For sMepS degradation assays, 7 μg of sMepS was incubated with 2 μg WT Prc or Prc mutant protein and 1 μg of sNlpI (at a 1:1 molar ratio) in each reaction mixture (10 μl). The reaction mixtures were incubated and processed as described above, except that the percentage of the bis-Tris protein gels used was 12% (Invitrogen).

### Viability assays.

Cultures grown overnight were serially diluted in minimal medium, 5 μl of each dilution was spotted on the plates indicated below, and the plates were incubated at the appropriate temperature overnight. The plates contained 1.5% agar in either LB (1% tryptone, 0.5% yeast extract, 1% NaCl) or LBON (1% tryptone, 0.5% yeast extract).

### AUC-SV.

Analytical ultracentrifugation sedimentation velocity (AUC-SV) analyses were performed in an XL-A analytical ultracentrifuge equipped with a 4-hole An-60 Ti rotor and 12-mm double-sector charcoal-filled Epon centerpieces (Beckman Coulter). Sedimentation velocity measurements, obtained using the absorbance optics of the reference buffer and samples in 20 mM Tris-HCl (pH 8.0), 150 mM NaCl, were carried out at 45,000 rpm and 20°C. The buffer density and viscosity were calculated by use of the Sednterp tool. Sedimentation coefficient (S) distributions were calculated using SEDFIT program and converted to 20°C and water conditions. AUC-SV results were normalized and plotted using GraphPad Prism (version 7) software (GraphPad Software, USA).

### DSF.

Purified PDZ and Prc mutant proteins were diluted in 20 mM Tris-HCl (pH 8.0), 150 mM NaCl buffer to a final concentration of 0.2 mg/ml. Fifteen microliters of diluted proteins was mixed with 1 μl 100× Sypro Orange (Sigma-Aldrich) and loaded in a LightCycler 480 Multiwell Plate 96 device (Roche) for the differential scanning fluorimetry (DSF) assay. The DSF assay was conducted on a LightCycler 480 II real-time PCR system (Roche), with the excitation and emission wavelengths being set to 465 and 580 nm, respectively. Fluorescence as a function of temperature was recorded from 20°C to 95°C at a rate of 0.01°C/s. Melting curves were exported for further processing with GraphPad Prism (version 7) software (GraphPad Software, USA).

### Limited proteolysis.

For the V8 proteolysis assay, 6 μg of Prc was incubated with 0.6 μg of V8 protease (Roche) in 0.1 M Tris (pH 7.4) at 25°C. Samples were incubated, and the reactions were stopped at different time points by adding 5× SDS-PAGE loading dye. After being heated at 98°C, samples were loaded onto a 4 to 12% bis-Tris gel. Protein bands were then detected and analyzed by Coomassie blue staining.

### Data availability.

The structures of unliganded Prc presented in the study have been submitted to the Protein Data Bank and may be found under accession numbers 6IQQ (NlpI-Prc-S452I/L252Y), 6IQR (Prc-S452I/L252Y), 6IQS (NlpI-Prc-L245A/L340G), and 6IQU (NlpI-PrcΔPDZ).
