# Supplementary figures and images for: Structural Basis for the Differential Regulatory Roles of the PDZ Domain in C-Terminal Processing Proteases
Source: mBio. 2019 Aug 6;10(4):e01129-19. doi: 10.1128/mBio.01129-19 (PMC6686036; doi:10.1128/mBio.01129-19)

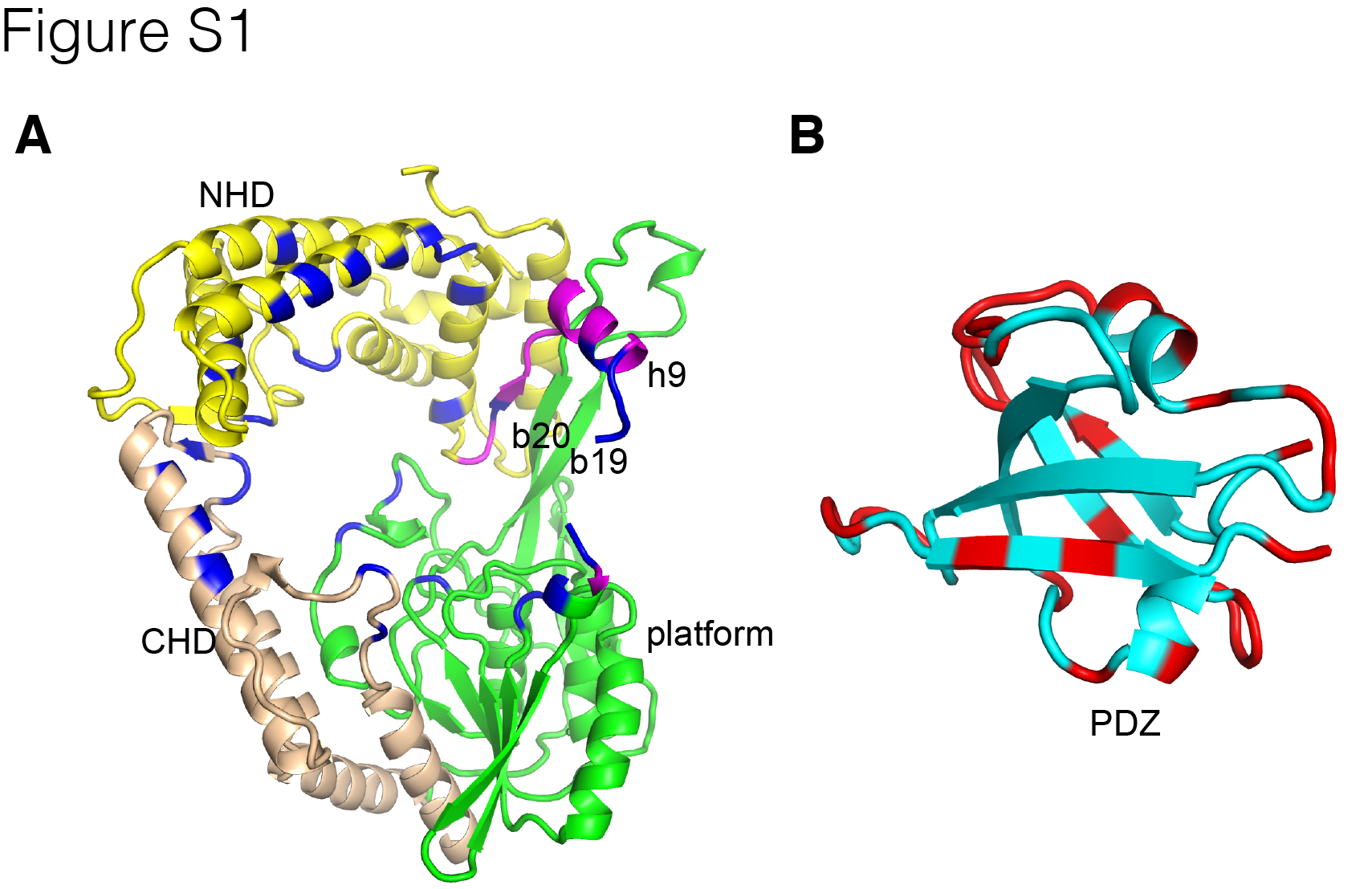

Supplement: FIG S1 [file mBio.01129-19-sf001.tif]

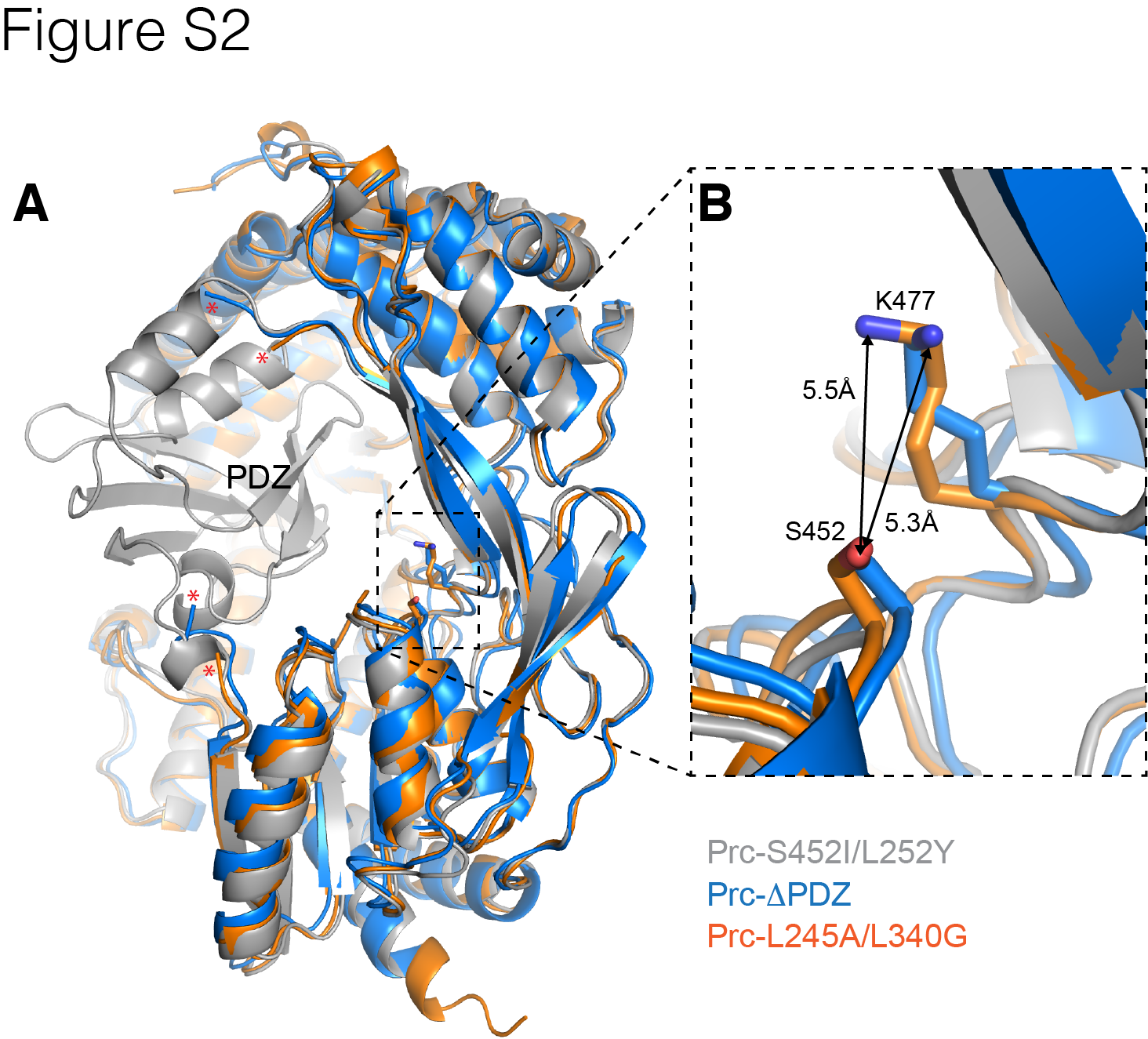

Supplement: FIG S2 [file mBio.01129-19-sf002.tif]

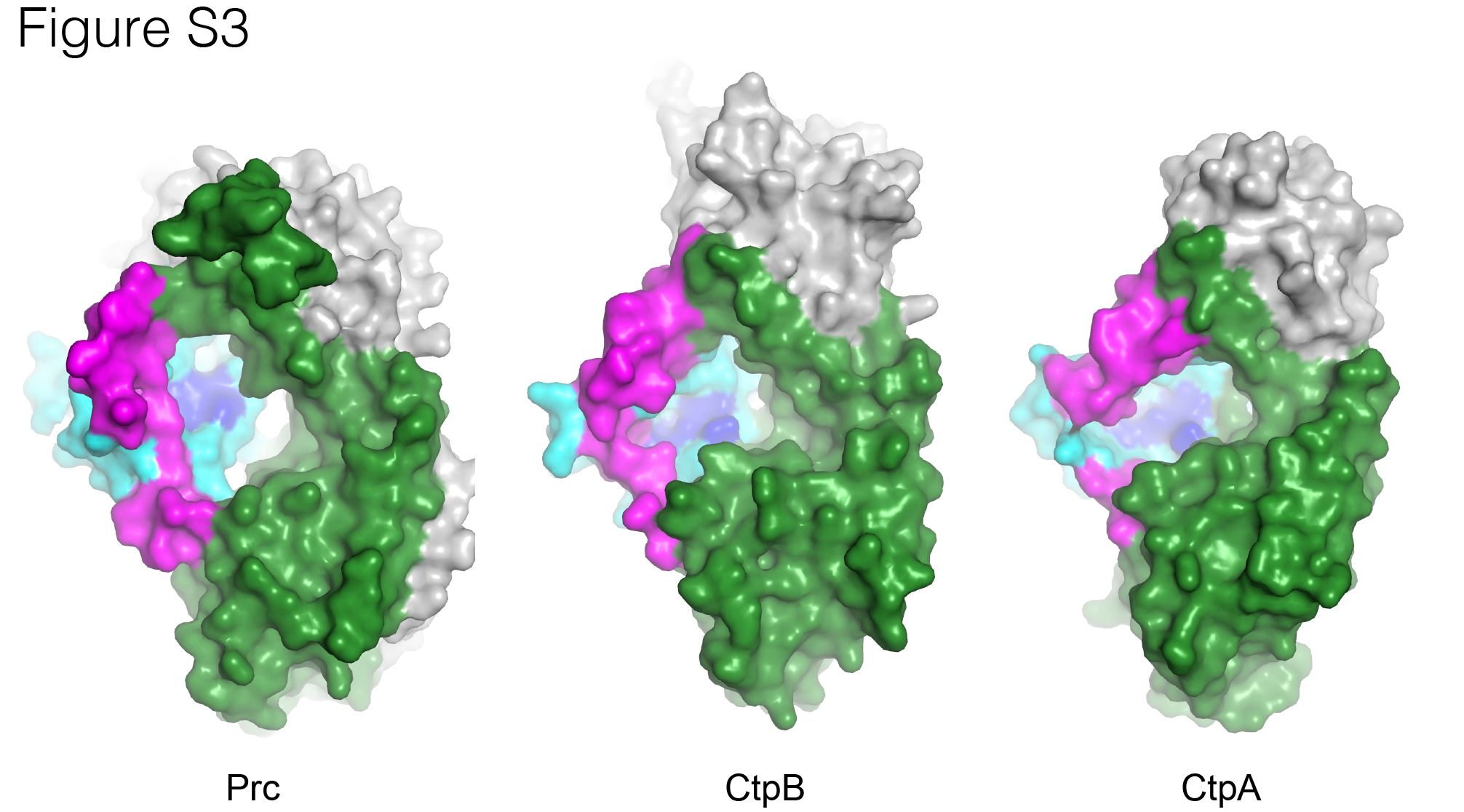

Supplement: FIG S3 [file mBio.01129-19-sf003.tif]

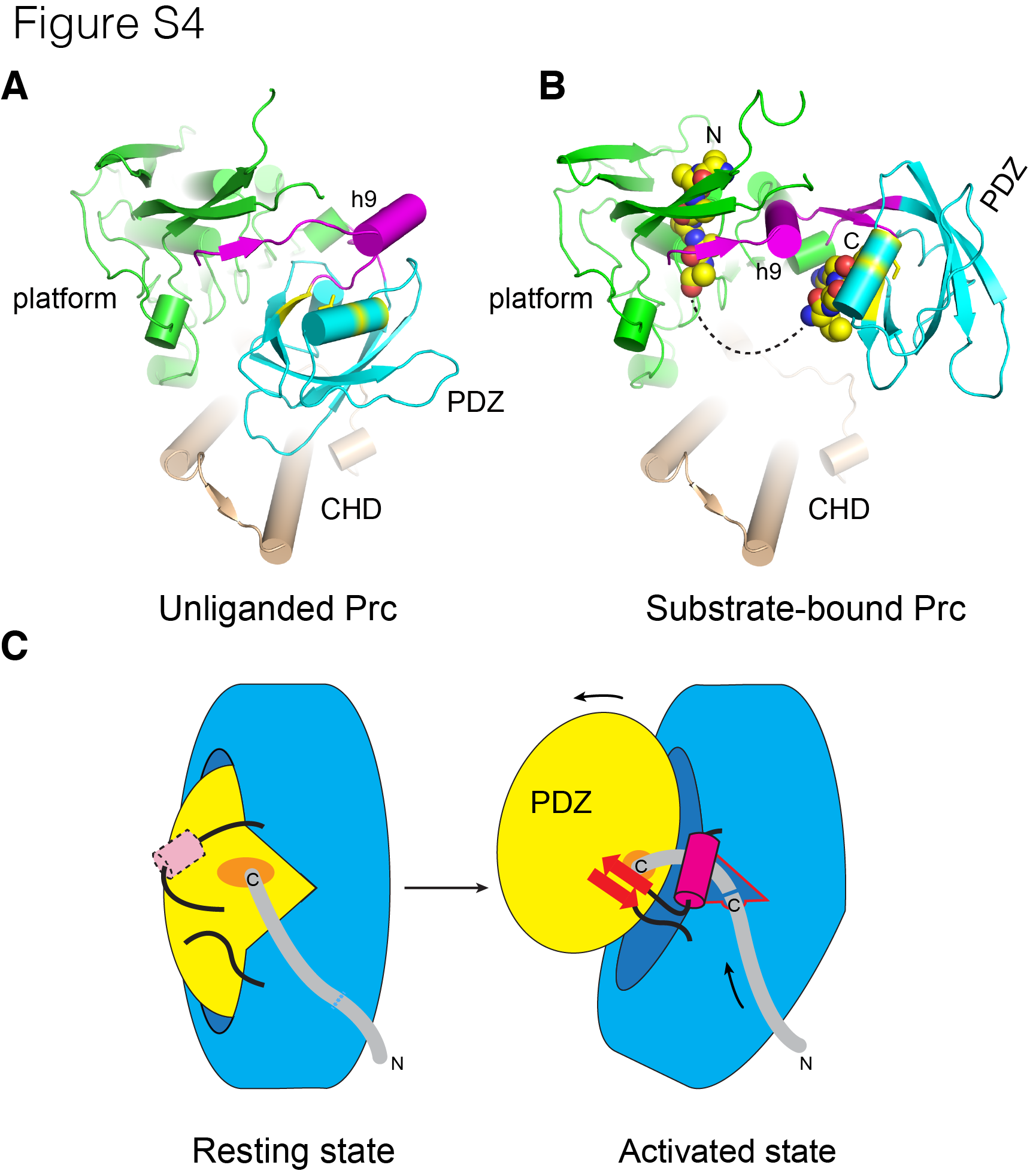

Supplement: FIG S4 [file mBio.01129-19-sf004.tif]
