# Supplementary material for: Structural Basis for the Differential Regulatory Roles of the PDZ Domain in C-Terminal Processing Proteases
Source: mBio. 2019 Aug 6;10(4):e01129-19. doi: 10.1128/mBio.01129-19 (PMC6686036; doi:10.1128/mBio.01129-19)
Supplement: TABLE S1 [file mBio.01129-19-st001.docx]

**Table S1**

|  | Prc-S452I/L252Y | NlpI-Prc-S452I/L252Y |
| --- | --- | --- |
| Space group | P3_2_21 | P2_1_2_1_2_1_ |
| Cell dimensions |  |  |
| a, b, c (Å) | 129.28,129,28, 236.59 | 118.94, 143.88, 153,59 |
| α, β, γ (˚) | 90,90,120 | 90, 90, 90 |
| Data collection |  |  |
| Wavelength (Å) | 1.00000 | 0.99984 |
| Resolution (Å) | 50~3.42 (3.54~3.42) | 30~2.80 (2.90~2.80) |
| Total observations | 136,067 | 509,147 |
| Unique reflections | 31,494 (3,117) | 64,129 (6,401) |
| R_means_ (%) | 14.5 (94.2) | 15.4 (74.8) |
| R_merge_ (%) | 7.5 (65.1) | 10.2 (61.5) |
| R_pim_^b^ (%) | 6.9 (44.6) | 5.5 (26.3) |
| Completeness (%) | 99.4 (100) | 98.3 (99.4) |
| I/σ(I) | 13.8 (2.1) | 13.9 (3.30) |
| Redundancy | 4.3 (4.5) | 7.9 (6.9) |
| Refinement |  |  |
| Resolution (Å) | 29.97~3.42 (3.50~3.42) | 29.98~2.80 (2.87~2.80) |
| R_work_/R_free_ (%) | 23.9/28.7 | 22.2/27.8 |
| No. of Reflections | 27,244 | 60,219 |
| No. atoms /B factor (Å2) |  |  |
| Protein | 101,143/74.06 | 14,407/56.12 |
| Water | 2/50.00 | 95/39.27 |
| Calcium ion |  | 2/53.09 |
| Rms deviations |  |  |
| Bond lengths (Å) | 0.014 | 0.012 |
| Bond angles (˚) | 1.95 | 1.88 |
| Ramachandran statistics (%) |  |  |
| most favored | 89.7 | 91.6 |
| additionally allowed | 9.0 | 8.1 |
| generously allowed | 0.9 | 0.3 |
| disallowed regions | 0.4 | 0.0 |
| disordered regions | chain B 533-541 | chain C 242-243  chain D 242-243, 324 |
| PDB code | 6IQR | 6IQQ |

**Table S1 (continued)**

|  | NlpI-Prc-L245A/L340G | NlpI-Prc-ΔPDZ |
| --- | --- | --- |
| Space group | P2_1_2_1_2_1_ | C2_1_2_1_2 |
| Cell dimensions |  |  |
| a, b, c (Å) | 119.34, 144.41, 152.50 | 105.83, 151.08, 148.38 |
| α, β, γ (˚) | 90, 90, 90 | 90, 90, 90 |
| Data collection |  |  |
| Wavelength (Å) | 1.10000 | 0.99984 |
| Resolution (Å) | 30~2.70 (2.80~2.70) | 50~2.90 (3.00~2.90) |
| Total observations | 351,114 | 111,108 |
| Unique reflections | 69,840 (6,187) | 25,531 (1,987) |
| R_means_ (%) | 9.8 (81.3) | 8.8 (27.6) |
| R_merge_ (%) | 6.4 (54.8) | 5.7 (20.1) |
| R_pim_ (%) | 4.2 (39.4) | 4.2 (14.1) |
| Completeness (%) | 94.7 (85.1) | 95.9 (76.0) |
| I/σ(I) | 13.4 (3.68) | 18.9 (4.4) |
| Redundancy | 5.0 (3.5) | 4.4 (3.4) |
| Refinement |  |  |
| Resolution (Å) | 29.91~2.69 (2.76~2.69) | 38.80~2.90 (2.98~2.90) |
| R_work_/R_free_ (%) | 20.5/24.5 | 19.2/23.3 |
| No. of Reflections | 65,488 | 22,490 |
| No. atoms /B factor (Å2) |  |  |
| Protein | 12,841/54.70 | 6434/53.81 |
| Water | 51/39.53 | 11/42.17 |
| Rms deviations |  |  |
| Bond lengths (Å) | 0.021 | 0.015 |
| Bond angles (˚) | 2.05 | 2.08 |
| Ramachandran statistics (%) |  |  |
| most favored | 92.0 | 89.3 |
| additionally allowed | 7.6 | 10.4 |
| generously allowed | 0.1 | 0.3 |
| disallowed regions | 0.3 | 0.0 |
| disordered regions | chain C 233-342  chain D 233-342 | Chain B 235-247 |
| PDB code | 6IQS | 6IQU |
