# Supplementary material for: Structural Basis for the Differential Regulatory Roles of the PDZ Domain in C-Terminal Processing Proteases
Source: mBio. 2019 Aug 6;10(4):e01129-19. doi: 10.1128/mBio.01129-19 (PMC6686036; doi:10.1128/mBio.01129-19)
Supplement: TABLE S2 [file mBio.01129-19-st002.docx]

**Table S2**

| Crystal structure | Chain | Two-chain r.m.s.d (Å)* | Cα atoms used for superposition |
| --- | --- | --- | --- |
| NlpI-Prc-S452I/L252Y | C | 0.92/1.16 | 587/637 |
| NlpI-Prc-S452I/L252Y | D | 0.82/1.08 | 591/636 |
| Prc-S452I/L252Y | B | 0.38/0.68 | 547/631 |
| NlpI-Prc-L245A/L340G | C | 0.60/1.51 | 480/529 |
| NlpI-Prc-L245A/L340G | D | 0.66/1.54 | 485/529 |
| NlpI-Prc-ΔPDZ | B | 1.04/1.88 | 519/534 |
| NlpI-Prc-S452I | C | 0.60/0.96 | 503/580 |
| NlpI-Prc-S452I | D | 0.56/1.78 | 491/564 |
