# Supplementary material for: Structural Basis for the Differential Regulatory Roles of the PDZ Domain in C-Terminal Processing Proteases
Source: mBio. 2019 Aug 6;10(4):e01129-19. doi: 10.1128/mBio.01129-19 (PMC6686036; doi:10.1128/mBio.01129-19)
Supplement: TABLE S3 [file mBio.01129-19-st003.docx]

**Table S3**

| **Primer Name** | **Sequence (5’ to 3’)** |
| --- | --- |
| sFtsI forward | TCT AGC TAG CAT GGG CGA CAT GCG TTC TCT TCG CG |
| sFtsI reverse | CGG GAT CCT TAC GAT CTG CCA CCT GTC CCC |
| Prc-L252Y forward | GAA GGT ATT GGC GCA GTG TAT CAA ATG GAT GAT GAC TAC |
| Prc-L252Y reverse | GTA GTC ATC ATC CAT TTG ATA CAC TGC GCC AAT ACC TTC |
| Prc-S452I forward | GTT GAC CGC TTC AGT GCT ATC GCT TCA GAA ATC TTT GCC |
| Prc-S452I reverse | GGC AAA GAT TTC TGA AGC GAT AGC ACT GAA GCG GTC AAC |
| Prc-L245A forward | ACT GAA ATG AGT TTG TCG GCT GAA GGT ATT GGC GCA GTG |
| Prc-L245A reverse | CAC TGC GCC AAT ACC TTC AGC CGA CAA ACT CAT TTC AGT |
| Prc-L340G forward | CGT GAA CGT ATT CGT GGC GAA GAC CGC GCG GTT |
| Prc-L340G reverse | AAC CGC GCG GTC TTC GCC ACG AAT ACG TTC ACG |
| Prc-∆PDZ forward | TGA GTT TGT CGC TGG AAT TCC TCG AAG ACC GCG CGG TTA A |
| Prc-∆PDZ reverse | TTA ACC GCG CGG TCT TCG AGG AAT TCC AGC GAC AAA CTC A |
| sNlpI forward | CAT CAT CAC AGC AGC GGC GAG AAT CTC TAC TTC CAG GGT CAT ATG AGT AAT ACT TCC |
| sNlpI reverse | GGA AGT ATT ACT CAT ATG ACC CTG GAA GTA GAG ATT CTC GCC GCT GCT GTG ATG ATG |
| sMepS-6xHis-C forward | GAT CCA TAT GAG TGC AAA TAA CAC CGC A |
| sMepS-6xHis-C reverse | GAT CCT CGA GTT AGT GGT GGT GGT GGT GGT GGC TGC GGC TGA GAA CCC G |
